# Impact of an Oral Hygiene Intervention in People with and without Dementia on Oral Health Parameters—Results from the Oral Health, Bite Force, and Dementia (OrBiD) Pilot Study

**DOI:** 10.3390/jcm11051356

**Published:** 2022-03-01

**Authors:** Julia Jockusch, Siri Nitschke, Werner Hopfenmüller, Oliver Schierz, Sebastian Hahnel, Ina Nitschke

**Affiliations:** 1 Gerodontology Section, Department of Prosthetic Dentistry and Materials Science, University of Leipzig, 04103 Leipzig, Germany; siri.nitschke@studserv.uni-leipzig.de (S.N.); oliver.schierz@medizin.uni-leipzig.de (O.S.); sebastian.hahnel@medizin.uni-leipzig.de (S.H.); or ina.nitschke@zzm.uzh.ch (I.N.); 2 University Research Priority Program “Dynamics of Healthy Aging”, University of Zurich, 8050 Zurich, Switzerland; 3 Institute of Biometry and Clinical Epidemiology, Charité—Universitätsmedizin Berlin, Corporate Member of Freie Universität Berlin, Humboldt-Universität zu Berlin, and Berlin Institute of Health, 10117 Berlin, Germany; werner.hopfenmueller@charite.de; 4 Clinic of General, Special Care and Geriatric Dentistry, Center of Dental Medicine, University of Zurich, 8032 Zurich, Switzerland

**Keywords:** oral hygiene intervention, dementia, DMFT, periodontal screening index, bleeding on probing, oral hygiene index, geriatric dentistry, old, cognitive impairment, oral health

## Abstract

This study aimed to assess the influence of an oral hygiene intervention on oral health, depending on the degree of dementia. A clinical evaluation of oral health parameters (index of decayed, missing, and filled teeth (DMFT-index), periodontal screening index (PSI), oral hygiene index (OHI), and bleeding on probing (BOP)) was performed in 120 subjects assigned to five groups, based on the mini mental state examination (MMSE) at baseline and after 12 months. Each MMSE group (no dementia (noDem, MMSE 28–30), mild cognitive impairment (mCI, MMSE 25–27), mild dementia (mDem, MMSE 18–24), moderate dementia (modDem, MMSE 10–17), and severe dementia (sDem, MMSE ≤ 9)) was split into control (no intervention) and experimental groups (intervention on oral hygiene: increased frequency, daily usage of high-fluoride toothpaste). In total, 99 out of 120 subjects were included in the analysis. The dropout rate was high in subjects with modDem and sDem due to death. In subjects with noDem, mCI, and mDem, no changes in the DMFT were found, but improvements in the OHI, BOP, and PSI were observed. Subjects with modDem or sDem demonstrated a deterioration in DMFT; however, in these patients, OHI improved in all control and experimental groups, BOP improved in the experimental group only, and PSI did not improve at all. The scope of improving oral health parameters by increasing the recall frequency and by continuously using high fluoride toothpaste is at its limits in people with severe dementia. Multidimensional approaches should be sought to improve the oral health of vulnerable older patients.

## 1. Introduction

The number of people suffering from dementia is increasing worldwide. By 2050, the prevalence is predicted to have doubled or even tripled in most industrialized countries [1]. Similarly, the proportion of people in need of care is increasing with a steadily growing older population [2]. Because of the prevention efforts of the last decades, old and very old people retain more of their teeth into old age today [3]. The decline in cognitive abilities, culminating in dementia, leads to an increase in the need for care. This trend is also observed in the field of dentistry. Here, at the same time, the reduced utilization of dental services can be observed [4]. However, patients with advanced dementia are not able to take care of their oral and denture hygiene on their own.

Often, residents in long-term care facilities (LTCF) suffer from poor oral health and oral hygiene, combined with general dental neglect and many oral diseases [5]. Higher incidence rates of dental caries, especially from retained roots and coronal and root caries [6], and periodontal disease leading to pain and other oral problems are reported in the literature [7,8,9,10,11]. Other studies showed that institutionalized older people, in particular, are at greater risk of being edentulous. Furthermore, higher numbers of decayed or missing teeth and poor periodontal status are identified in both institutionalized and non-institutionalized older people [12], and in older people with and without dementia [13]. Moreover, the increase in age and the associated multimorbidity shows that dentist visits are less frequently utilized [14]. Especially in people with dementia, a significant reduction in utilization behavior can be observed. This results in an increasing number of decayed teeth and a decrease in the degree of restoration with increasing cognitive impairment or dementia. Additionally, oral/denture hygiene deteriorates significantly [13].

Furthermore, the oral health awareness of caregivers in LTCF is not reflected in adequate oral health care for patients. There is a gap between the positive oral health-supporting attitudes of the caregivers and the adequate and preventive behavior of the staff concerning oral health and the actual oral health care of the patients [15]. This is mostly due to a lack of knowledge and the psychological barriers involved in working within the oral cavity of another person [15,16,17]. In addition, the need for treatment is often underestimated or wrongly determined by caregivers [18,19]. This often results in diseases of the oral hard and soft tissues. In these cases, therapy becomes more difficult with increasing cognitive impairment and is sometimes no longer possible without treatment being conducted under general anesthesia [20].

As oral hygiene seems to be a major contributor to the quality of life of older people and may also prevent aspiration pneumonia and reduce the associated risk of death, its maintenance becomes even more important [21]. There is little evidence in the literature showing that oral health education interventions for caregivers or residents are an effective way to improve oral health in people with and without dementia who are living in LTCF [22,23].

It can be assumed that an increase in recall frequency (dental visits and professional tooth- and denture-cleaning) can help to improve oral health. It must be clarified to what extent this effect depends on the degree of dementia. Therefore, this study aimed to assess the influence of intervention in oral health (increase in the recall frequency of professional oral hygiene care) depending on the degree of dementia. The authors hypothesized that an increase in the utilization of dental services (an increase in the recall frequency) has a positive influence on the oral health-related parameters (index of decayed, missing, and filled teeth (DMFT-Index), periodontal screening index (PSI), bleeding on probing (BOP), and oral hygiene index (OHI)) of people with dementia.

## 2. Materials and Methods

The data of this analysis are part of the OrBiD (“Oral Health, Bite Force and Dementia”) (ClinicalTrials.gov NCT03775772) pilot study [13,24]. In total, 120 subjects were assigned to one of five evaluation groups with different degrees of dementia using the mini mental state examination (MMSE) [25] (MMSE-groups: 1: no dementia (noDem, MMSE 28–30); 2: mild cognitive impairment (mCI, MMSE 25–27); 3: mild dementia (mDem, MMSE 18–24); 4: moderate dementia (modDem, MMSE 10–17); 5: severe dementia (sDem, MMSE ≤ 9)). The MMSE is employed to test verbal and non-verbal episodic memory, orientation in time and place, and visual-constructive skills (to a maximum of 30 points). The MMSE was performed by a dentist with all subjects who were without a medical diagnosis of dementia or who had not provided information on the MMSE score in their requested medical reports. Since dementia was expected to have a major influence, stratified randomization was used. In a first step, all participants were assigned to one of the five MMSE groups according to their MMSE score. In a second step, within the MMSE groups, further assignment to the experimental group (*n* = 12) or to the control group (*n* = 12) of the study part analyzed here was conducted randomly, based on the order of study participation (not influenceable but instead according to registration for study participation and appointment by uninvolved persons) within MMSE groups 1–5 (even numbers—experimental group, odd numbers—control group).

### 2.1. Eligibility Criteria

Subjects either living at home or in LTCF, who were aged 60 years and older, were included regardless of their cognitive abilities. A prerequisite was sufficient knowledge of German to be able to participate in an interview and to follow the intervention instructions during the evaluations and the intervention appointments. Study participation for subjects with acute dental problems (e.g., pain, abscesses, etc.) requiring emergency treatment started after successful emergency treatment. Before the start of the study, all subjects should have had no or a maximum of one dental hygiene session within the previous 12 months. Subjects with physical limitations in the upper body (e.g., musculoskeletal or neuromuscular conditions, such as paralysis of the arms, arthritis, conditions after stroke with the associated impairment of motor skills, and facial nerve paralysis) were excluded.

### 2.2. Study Intervention

An instruction to intensify daily individual and professional oral and denture hygiene was given to all subjects in the experimental groups. The instruction included a recommendation to intensify oral hygiene at home, including the usage of a high-fluoride-content toothpaste (Duraphat^®^ 5000 ppm, Colgate™; GABA Switzerland, Therwil, Switzerland) at least twice a day instead of the toothpaste previously used and the recommendation not to rinse, but only to spit out after brushing. The use of additional aids (e.g., mouth rinses) was not advised. The procedure was explained directly to the subjects orally if they were cognitively able to perform their daily oral hygiene at home independently. In the case of subjects requiring assistance with daily oral and denture hygiene, or where a third person performed oral and denture hygiene, the procedure was explained to the persons involved, such as relatives, legal representatives, or caregivers.

Additionally, the frequency of professional tooth and denture cleaning was increased (four appointments for professional oral and denture hygiene at intervals of 3 months within a year). Tooth cleaning was carried out by the use of manual instruments or ultrasonic attachments (no use of airflow devices, etc., as this was not possible in the LTCF). Denture cleaning was performed using an ultrasonic cleaning bath.

Subjects in the control groups did not receive an oral hygiene intervention but were encouraged to continue their daily oral hygiene as usual. All subjects, whether in the experimental or control group, had access to dental treatment at all times, regardless of study participation.

### 2.3. Measurements

All clinical procedures and evaluations were performed by a single investigator, either in a large dental office (community-dwelling, non-institutionalized subjects) or in the LTCF (institutionalized subjects) in the canton of Zurich, Switzerland.

All subjects in the control and experimental groups were evaluated (before the start of the intervention for subjects of the experimental groups) at the beginning of the study (baseline) and 12 months after the baseline evaluation (final).

The following parameters were evaluated: sociodemographic data (age, sex, living situation (community-dwelling vs. LTCF)), the mini-nutritional assessment (MNA) [26], and the oral functional capacity (OFC) [27] were recorded for all subjects. The DMF/T-Index [28,29] (D: decayed; M: missing; F: filled; T: teeth) as a measure of caries experience was used. The periodontal screening index (PSI) [30] was recorded as the worst value per sextant. Bleeding on probing (BOP) as an indicator of the presence of bleeding, caused by gentle tissue manipulation at the depth of the gingival sulcus or the gingiva-to-tooth interface, was evaluated. The oral hygiene index (OHI) [31] was used to evaluate and classify oral hygiene (Supplementary Material S1).

### 2.4. Statistical Analysis

A power calculation was not performed for this pilot study because no literature on the endpoints was found. The sample size was determined based on similarly designed studies and the sample sizes used therein [32,33].

Since this study was a pilot study, the statistical analyses corresponded. Within the longitudinal component of the study, the effects of the intervention on oral hygiene have been compared. Descriptive and graphical tools were used where appropriate. Additionally, mixed linear models were tentatively used to account for the temporal dependency structure and to control for age and gender. Robust non-parametric tests have been used for the comparison between mental state and intervention effect if the parametric model assumptions were violated. In order to account for potential deviations of the research protocol, intention-to-treat (ITT) and per-protocol (PP) analyses were performed. Subjects lost to dropout were replaced with new recruits. Missing values, resulting from subjects who were not able to undergo a complete examination, were estimated by statistical means with a multivariate imputation algorithm.

To visualize the influence of the intervention or the absence of the intervention on the outcome variables, differences (delta) were calculated between the evaluation time points (DMFT-Index and OHI-Score/DI/CI: final minus baseline; PSI and BOP: baseline minus final). Additionally, for the delta PSI value, the quadratic weighting of the PSI code for baseline and final was done before calculation. Statistical analysis was performed using SPSS Version 23 [34].

### 2.5. Ethical Consideration

The study was approved by the competent cantonal ethics committee of Zurich (KEK-ZH 2017-00363). All subjects or their legal representatives gave written informed consent.

## 3. Results

In total, 99 out of 120 subjects were included in the analysis (noDem *n* = 24; mCI *n* = 24; mDem *n* = 23; modDem *n* = 17; sDem *n* = 11). All dropouts (*n* = 21) resulted from the death of the subjects. As a result of the COVID-19 pandemic, the final evaluations of some subjects living in LTCF were conducted 4–8 weeks later than planned. Various subjects had also died during this period of time (Table 1). Table 1 shows the data on sociodemographic items (age, sex, living situation) for all subjects.

Oral functional capacity (OFC) and its parameters became worse with the increase in the degree of dementia in all subjects. Furthermore, the malnourished nutritional status increased with increasing dementia, independently from the control or experimental groups (Table 1).

### Longitudinal Alterations of the Outcome Parameters

**Figure 1:**
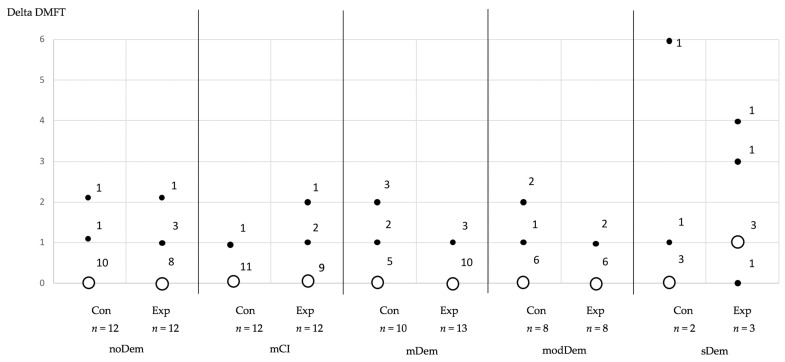
Changes in the DMFT index over time (final minus baseline), shown as ΔDMFT, separated by the mini mental state examination (MMSE) group and subgroup (control and experimental group). The legends at the points indicate the number of subjects with the corresponding ΔDMFT value. (Con: control group; Exp: experimental group) (MMSE groups—noDem: no dementia; mCI: mild cognitive impairment; mDem: mild dementia; modDem: moderate dementia; sDem: severe dementia) (hollow circles correspond to the majority of counts of changes in each evaluation group, filled circles demonstrate minor amount of counts of changes in each evaluation group, numbers at hollow and filled circle correspond to number of changes).

The DMFT values were highly distributed in all MMSE groups, except the control group of the MMSE group with sDem (Table 2). The majority of subjects in the control and experimental cohorts of all MMSE groups exhibited no change in the DMFT index between baseline and final evaluation (Δ DMFT). Only those subjects in the experimental group with sDem showed a deterioration in the DMFT index in the longitudinal analysis (Table 2, Figure 1).

**Figure 2:**
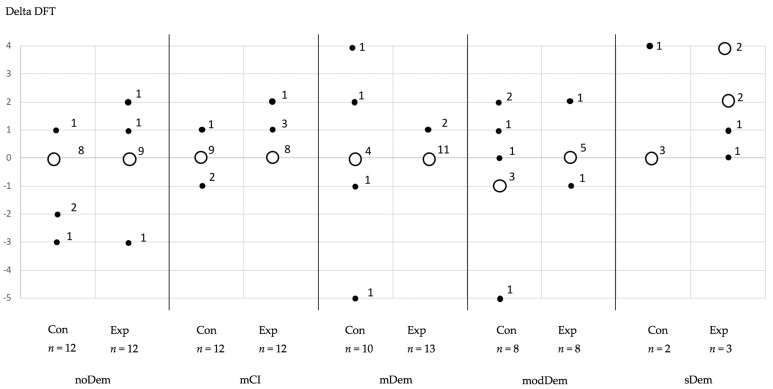
Changes in the DFT index over time (final minus baseline), shown as ΔDFT, separated by mini mental state examination (MMSE) group and subgroup (control and experimental group). The legends at the points indicate the number of subjects with the corresponding ΔDFT value. (Con: control group; Exp: experimental group) (MMSE groups—noDem: no dementia; mCI: mild cognitive impairment; mDem: mild dementia; modDem: moderate dementia; sDem: severe dementia) (hollow circles correspond to the majority of counts of changes in each evaluation group, filled circles demonstrate minor amount of counts of changes in each evaluation group, numbers at hollow and filled circle correspond to number of changes).

In terms of the delta DFT values, only minor differences were observed between the MMSE groups. Subjects of the experimental group with sDem predominantly exhibited deterioration (an increase in the DFT value) (Table 2, Figure 2).

**Figure 3:**
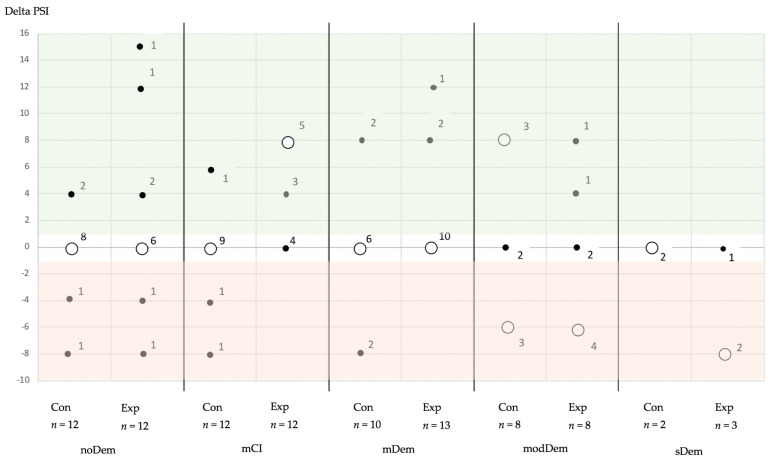
Changes in the periodontal screening index (PSI—code 0: healthy; code 1/2: gingivitis/calculus present; code 3: moderate periodontitis; code 4: severe periodontitis) over time separated by mini mental state examination (MMSE) group and subgroup (Con: control group; Exp: experimental group) after calculation of the quadratic weighted PSI code for baseline and final (ΔPSI = baseline minus final) (color coding—red: deterioration over time; white: no change; green: improvement) (MMSE groups—noDem: no dementia; mCI: mild cognitive impairment; mDem: mild dementia; modDem: moderate dementia; sDem: severe dementia) (hollow circles correspond to the majority of counts of changes in each evaluation group, filled circles demonstrate minor amount of counts of changes in each evaluation group, numbers at hollow and filled circle correspond to number of changes).

For delta PSI, an improvement in the PSI code was observed more often for the experimental groups of the MMSE groups with noDem, mCI, and mDem, compared to the control groups. This phenomenon was not observed for the MMSE groups with modDem and sDem (Table 3, Figure 3).

**Figure 4:**
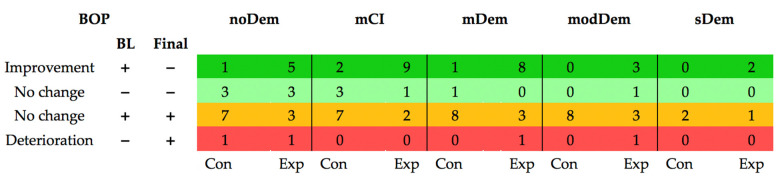
Changes in bleeding on probing (BOP) over time, separated by mini mental state examination (MMSE) group and subgroup (Con: control group; Exp: experimental group) (green: improvement; light-green and orange: no change; red: deterioration) (Dementia groups—noDem: no dementia; mCI: mild cognitive impairment; mDem: mild dementia; modDem: moderate dementia; sDem: severe dementia). (BL: Baseline) (colors green to red symbolize the deterioration in BOP).

BOP was reduced in most subjects in the experimental groups of all MMSE cohorts, while all control groups showed a deterioration in BOP over time (Figure 4).

**Figure 5:**
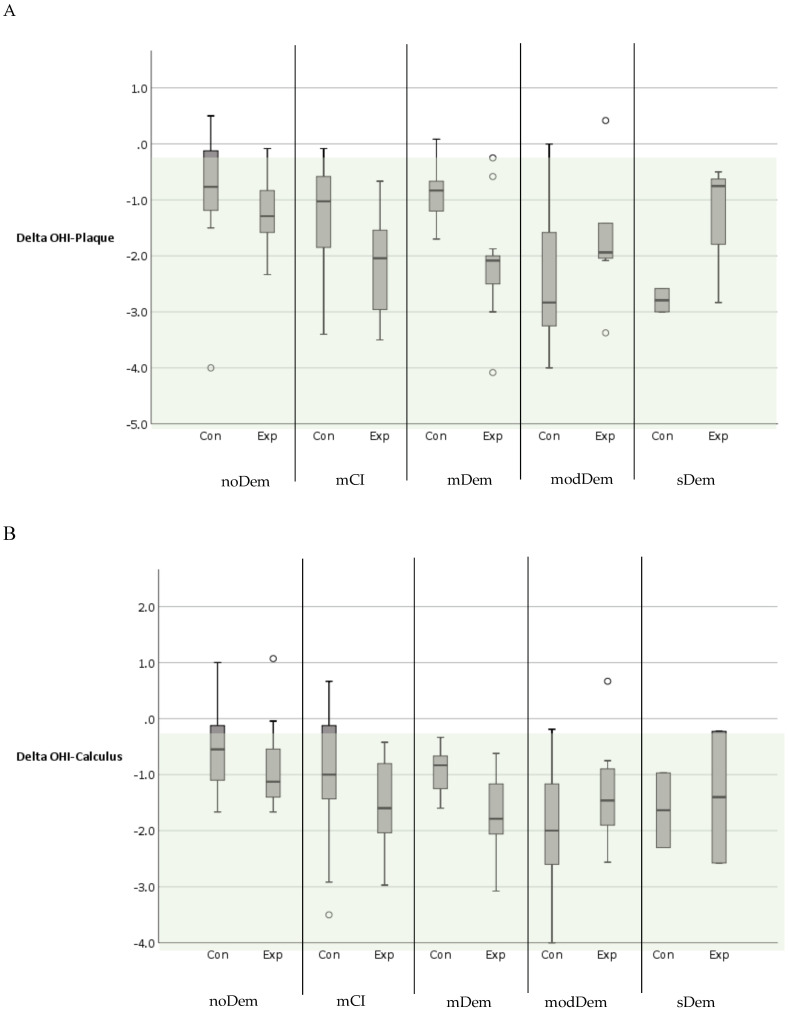

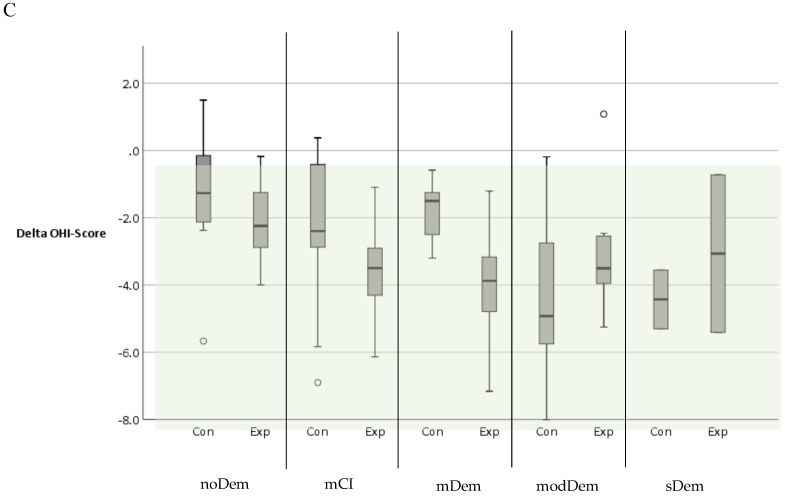
Changes in the oral hygiene index (OHI) and its components (DI: debris index; CI: calculus index) over time as: (**A**) ΔOHI Plaque; (**B**) ΔOHI Calculus; and (**C**) ΔOHI Score separated by mini mental state examination (MMSE) group and subgroup (control/experimental group) (Con: control group; Exp: experimental group) (MMSE group—noDem: no dementia; mCI: mild cognitive impairment; mDem: mild dementia; modDem: moderate dementia; sDem: severe dementia) (color coding—green: improvement in score; circle correspond to statistical outliers).

In the majority of subjects, regardless of their MMSE group or membership in the control or experimental group, an improvement was observed in the OHI score (Δ OHI Score) and in the individual OHI components DI (Δ OHI Plaque) and CI (Δ OHI Calculus). In the MMSE groups with noDem, mCI, and mDem, subjects in the experimental groups showed a stronger improvement in Δ OHI Plaque, Δ OHI Calculus, and Δ OHI Score than subjects in the corresponding control groups. For subjects in the MMSE group with modDem and sDem, this difference was not observed (Table 4, Figure 5A–C).

## 4. Discussion

In this part of the “Oral Health, Bite Force, and Dementia” (OrBiD) pilot study, an intervention regarding oral hygiene care (an increase in recall frequency (dental visits and professional tooth- and denture-cleaning)) was established for subjects in the experimental groups, while subjects in the control groups received no oral hygiene intervention.

The authors faced several challenges related to the sample of subjects in this study. The cooperation and compliance of people with dementia depend, on the one hand, on the individual and, on the other, on the daily cognition level of the patient. However, since cooperation was a decisive factor in the implementation of this intervention, the question arises as to what extent the results were influenced by reduced adherence to dental hygiene in people with moderate and severe dementia. In the literature, a procedure is described that should make it easier to carry out oral hygiene practices in people with dementia. The MOUTH program (“Managing Oral Hygiene Using Threat Reduction”), a relationship-based intervention, aims to create a clinically realistic approach to managing refusal behavior [35]. No such method was used in the present study. However, all examinations and treatments were performed by experienced specialists appointed by the German Association of Gerodontology (DGAZ). Furthermore, the recruitment of people with dementia and the simultaneous, underlying necessary interdisciplinary cooperation with the management, physicians, and caregivers of the LTCF present challenges that have often been described as complex [36]. A further aggravating factor is the high dropout rate in this patient population, which we attempted to counteract in the present study by re-recruitment. However, the target number of subjects per subgroup could not be reached, especially in the case of subjects with advanced dementia, as the mortality rate was further negatively affected by the COVID-19 pandemic and the resulting postponement of the final evaluation.

### 4.1. Limitations of the Study

The results of the current study could be subject to bias because the subjects or caregivers who performed daily oral hygiene were informed about the study objective. Therefore, it cannot be excluded that a bias may be present in subjects in both groups because of increased efforts in their oral hygiene. Consequently, it should also be considered in further studies that, in addition to professional oral and denture hygiene, the influence of daily oral hygiene should be considered. Data on adherence to oral hygiene measures that might have affected intervention outcomes could not be collected in this study for organizational, financial, and personnel reasons. In addition, caregivers may have made more effort to perform oral hygiene in all subjects during the observation period because this study was organized in cooperation with the home management and the responsible medical practitioner and was also reinforced by the repeated presence of the dental team. This circumstance could explain the lacking or only slight differences in certain oral health parameters between the control and experimental groups. It is also unclear whether an effect can be really shown in people with moderate and severe dementia since the implementation of the intervention was, in some cases, very limited and, in many cases, no comparable situation could be established concerning the treatment side (dental chair vs. chair in LTCF, with participants mostly sitting upright, etc.).

If there was no medical diagnosis of dementia or if it was not evident from the requested medical reports, one single dentist performed the MMSE test. An additional professionally qualified physician would have been the ideal solution, but this was not possible due to financial and personnel constraints. The dentist involved in the study is a specialist in seniors’ dentistry. The dentist was certified by the German Society for Gerodontology. In the course of this specialization, the participants are trained in geriatric assessment tools and are also taught how to perform the MMSE test.

Since the present study was designed to test the effects of an intervention that could be integrated into everyday clinical practice, the authors decided to use a modified classification of dementia severity, based on the MMSE scores described by Perneczky et al. [37]. The basis for setting the arbitrary thresholds was an expert panel that adopted a score range of the MMSE of 16–20 as the threshold for the impairment of everyday life by cognitive deficits.

The investigator was not blinded. However, the authors exclude a bias because clinical guidelines exist for all parameters collected and a subjective assessment can, therefore, be excluded to a high degree.

The assessment of non-institutionalized patients was performed in the large dental office, while the evaluation of care-dependent subjects was carried out in the LTCF. Attempts were made to create similar conditions for the assessments by using appropriate aids in the LTCF (e.g., head restraints, magnifying glasses with additional LED light).

### 4.2. Discussion of the Results

Due to the limitations of the study period, not all parameters collected may show measurable changes between evaluation time points. Time is an important factor in the development of carious lesions, their therapy, or the progression of periodontal disease. Therefore, there may have been an underestimation of the changes over time within the analysis.

DMFT and DFT values showed no significant differences between the control and experimental groups of all MMSE groups at the end of the study. Moreover, no significant changes between the baseline and final evaluation in the DMFT and DFT values within all MMSE groups were identified, except in the case of DMFT in subjects with sDem in the control group and DFT in subjects with sDem in the experimental group. The intervention appears to have had no effect on DMFT and its individual components in subjects with noDem to modDem. Overall, only patients with sDem experienced a deterioration in this oral health parameter.

An improvement of periodontal disease (i.e., reduction in the PSI code) was observed in subjects with noDem, mCI, and mDem in the experimental groups. This effect could not be achieved in the corresponding control groups of these MMSE groups. The influence of the intervention on the PSI could not be identified for subjects with modDem and sDem, although the BOP improved, as was the case in all other experimental groups in all MMSE groups. This observation may be explained by the lower adherence of patients with the intervention or by the higher PSI values in these subjects at baseline. It is easier to achieve changes in low PSI codes (1–2) than to reduce PSI 4 to lower values.

The OHI and its components, DI and CI, improved in all subjects in the study during the observation period. Subjects with noDem, mCI, and mDem in the experimental groups showed a stronger improvement than subjects who did not receive an intervention. Again, the bias described above (no blinding of subjects and caregivers with respect to the study objective) could be responsible. Nevertheless, the intervention seems to have had an additional positive influence on these parameters.

Overall, subjects with modDem and sDem showed a deterioration in the oral health parameters DMFT/DFT and PSI, independent of the intervention. For the OHI and its components, subjects with modDem and sDem also exhibited an improvement, regardless of whether they participated in the intervention. This phenomenon supports previous research showing that people with dementia have a higher incidence of caries, periodontal disease, pain, and other oral problems [7,8,9,10,11].

The intervention of the study included the implementation of professional oral and denture hygiene at an increased frequency (four times per year) by a dental hygienist and the daily use of Duraphat^®^ 5000 ppm toothpaste (Colgate™) by the subjects of the experimental groups. In the literature, it is specified that dental hygienists have the skills and abilities to perform oral hygiene care and also to identify the majority of residents who should be treated by a dentist. Furthermore, they are able to develop appropriate hygiene concepts for residents in care facilities [38]. Therefore, the authors conclude that the use of dental hygienists to perform the intervention under dental supervision is appropriate.

It has been shown that the use of high-fluoride-content toothpaste (5000 ppm F, twice daily) in adults has a significant positive effect on the surface hardness of untreated root caries lesions [39]. This phenomenon may explain why no changes in DMFT or DFT index were observed in subjects with noDem to modDem in this study. It should also be mentioned that subjects with advanced dementia were mainly recruited in LTCF and, due to the implementation of a mobile dental concept (mobiDent™; Zurich, Switzerland), the use of Duraphat toothpaste once a week may also have occurred in subjects in the control groups during the observation period. This might also explain the lack of difference in these parameters between the control and experimental groups. However, in people with severe dementia, a deterioration in the DMFT/DT index was observed, regardless of participation in the intervention, which may be due to both the lack of compliance with the intervention and the frequent refusal to comply with daily oral and denture hygiene in general.

### 4.3. Future Perspective

There is currently no study in the literature with a comparable intervention in patients with and without dementia. Rather, the effectiveness of oral hygiene education programs in caregivers is often discussed.

Studies point out the limited evidence for the effectiveness of the oral health education of caregivers to improve oral health in people with dementia [22,23]. For example, the implementation of a standardized oral care program (teaching, instruction on the use of individualized oral care concepts, and the training of caregivers to perform oral hygiene in patients with refusal behavior) did not lead to a long-term reduction in the incidence of pneumonia [40]. Otherwise, a situational learning perspective [36], the usage of a comprehensive, practically oriented program [41], or the use of oral and written instructions concerning oral and denture hygiene [42] can support both caregivers and residents in achieving adequate oral and denture hygiene. Other surveys point out the importance of early diagnosis of oral diseases and their prevention [43,44,45,46].

The literature also discusses a multidimensional approach based on individual needs as a concept for improving the oral health of vulnerable older adults [42]. In this context, preventive strategies that are applied to healthy older people should also be used for people with dementia [21]. This should include the implementation of preventive dental measures on a day-to-day basis, for both patients receiving outpatient care and in LTCF [42].

Future programs should include the following components, and their effectiveness should be evaluated in future studies:

- Ensuring that the dentist is called upon in cases of reduced mobility and cases of incipient vulnerability, by implementing mobile dental concepts;
- Referral of patients with an initial diagnosis of dementia by the physician to the dentist, enabling the early admission of older vulnerable patients for dental care and treatment including prevention;
- Educational programs for relatives on the topic of oral health in old age—as well as its relevance in dementia;
- Strengthening of interdisciplinary cooperation with medical doctors, LTCF, nutritionists, etc., and the establishment of multidisciplinary health care teams [47];
- A combination of comprehensive theoretical and practical training of nurses (according to the concept of “teach the teacher”) and relatives, thus enabling a reduction in psychological barriers to oral care;
- Reduction in barriers to the implementation of training concepts in the long-term care sector and an increase in financial support for the implementation of support measures, both from the state and from other cost units (e.g., health insurance companies);
- Improvement of evaluation programs, which are used in the care sector to estimate the need for care, to enhance dental problem detection [18];
- Prompt admission of patients when a need for treatment is diagnosed, to avoid anesthesia;
- Minimum 6-monthly dental recalls and the professional cleaning of teeth and dentures [47];
- Improvement of individual oral hygiene at home by setting up individual oral care programs (e.g., the use of toothpaste with a high fluoride content, etc.).

## 5. Conclusions

An increase in the dental recall intervals for professional oral and denture hygiene did not coincide with changes in the DMFT index in patients without dementia, up to mild dementia. Subjects with modDem and sDem exhibited a deterioration in this oral health parameter. The OHI and its components, DI and CI, improved in all subjects in the study during the observation period. Subjects with noDem, mCI, and mDem in the experimental groups exhibited greater improvement than subjects who did not receive an intervention. Subjects with modDem and sDem also showed improvement, regardless of whether they participated in the intervention. A reduction in the PSI was achieved in subjects with noDem, mCI, and mDem in the experimental groups as a result of the intervention. This effect was not observed in subjects with modDem and sDem, although the BOP did improve, as was the case in all other experimental groups in all MMSE groups.

## 6. Clinical Relevance

The scope of improving oral health parameters by increasing the recall frequency and continuously using high-fluoride toothpaste is very limited in people with severe dementia. Multidimensional approaches should be sought to improve the oral health of vulnerable older patients. A future program should always include the early identification of the patient’s geriatric oral health transition phase. Depending on the patient’s transition phase, oral health competence should then be increased, initially by the patient and later by the patient’s supportive environment, with a combination of concepts for individual daily oral and denture hygiene, professional oral and prosthetic care, and close-meshed control-oriented dental visits.

## Figures and Tables

**Table 1:** Sociodemographic data (age, gender, living situation), mini mental state examination (MMSE), data on nutritional status (MNA: mini nutritional assessment) and oral functional capacity (OFC), its parameters: therapeutic capability, oral hygiene ability, and self-responsibility, and the resulting resilience capacity level (RCL) (evaluation time point—BL: baseline; Final (12 months after BL)) (Exp: experimental group; Con: control group) (MMSE groups—noDem: no dementia; mCI: mild cognitive impairment; mDem: mild dementia; modDem: moderate dementia; sDem: severe dementia) (LTCF: long-term care facility).

Item	noDem				mCI				mDem				modDem				sDem			
	Con (*n* = 12)		Exp (*n* = 12)		Con (*n* = 12)		Exp (*n* = 12)		Con (*n* = 10)		Exp (*n* = 13)		Con (*n* = 9)		Exp (*n* = 8)		Con (*n* = 5)		Exp (*n* = 6)	
Sex (female) (*n*)	7		6		8		8		6		12		6		6		4		5	
Age (Median (Range))	74.5 (63–83)		76 (62–92)		78 (65–94)		83 (61–95)		85.5 (65–95)		82 (71–93)		91 (76–99)		86 (61–98)		78 (67–94)		90.5 (86–93)	
MMSE (Median (Range))	29 (28–30)		29 (28–30)		26.5 (25–27)		27 (25–27)		21.5 (18–24)		21 (18–24)		13 (10–17)		12.5 (10–17)		5 (1–9)		3.5 (0–9)	
Living situation (*n*)																				
Community-dwelling	9		12		7		7		2		2		0		0		0		0	
LTCF	3		0		5		5		8		11		9		8		5		6	
MNA (*n*)	BL	Final	BL	Final	BL	Final	BL	Final	BL	Final	BL	Final	BL	Final	BL	Final	BL	Final	BL	Final
	*n* = 12	*n* = 12	*n* = 12	*n* = 12	*n* = 12	*n* = 12	*n* = 12	*n* = 12	*n* = 10	*n* = 10	*n* = 13	*n* = 13	*n* = 7	*n* = 8	*n* = 8	*n* = 6	*n* = 5	*n* = 5	*n* = 5	*n* = 6
Normal (24–30)	10	10	10	11	8	9	7	10	5	6	4	3	0	0	1	0	0	0	0	0
At risk (17–23.5)	1	2	2	1	4	3	5	2	5	3	7	9	5	4	5	5	3	3	2	2
Malnourished (<17)	1	0	0	0	0	0	0	0	0	1	2	1	2	4	2	1	2	2	3	4
Oral functional capacity (*n*)	BL *n* = 12	Final *n* = 12	BL *n* = 12	Final *n* = 12	BL *n* = 12	Final *n* = 12	BL *n* = 12	Final *n* = 12	BL *n* = 10	Final *n* = 10	BL *n* = 13	Final *n* = 13	BL *n* = 9	Final *n* = 9	BL *n* = 8	Final *n* = 8	BL *n* = 5	Final *n* = 5	BL *n* = 6	Final *n* = 6
Therapeutic capability (*n*)																				
Normal	10	10	9	9	6	5	6	5	1	1	0	0	0	0	0	0	0	0	0	0
Slightly reduced	2	2	3	3	6	6	6	7	5	2	2	3	0	0	1	0	0	0	1	0
Greatly reduced	0	0	0	0	0	1	0	0	4	7	10	8	8	7	6	6	2	1	1	0
None	0	0	0	0	0	0	0	0	0	0	1	2	1	2	1	2	3	4	4	6
Oral hygiene ability (*n*)																				
Normal	8	9	8	9	3	3	2	2	1	1	0	0	0	0	0	0	0	0	0	0
Slightly reduced	4	3	4	3	8	8	7	8	3	2	3	4	0	0	1	0	0	0	0	0
Greatly reduced	0	0	0	0	1	1	3	2	6	5	7	6	8	5	5	5	2	1	1	0
None	0	0	0	0	0	0	0	0	0	2	3	3	1	4	2	3	3	4	5	6
Self-responsibility (*n*)																				
Normal	11	10	12	12	9	9	7	6	2	2	0	0	0	0	0	0	0	0	0	0
Reduced	1	2	0	0	3	2	5	6	5	1	5	6	4	2	3	0	0	0	0	0
None	0	0	0	0	0	1	0	0	3	7	8	7	5	7	5	8	5	5	6	6
Resilience capacity level (*n*)																				
Normal	8	8	6	8	3	2	2	2	1	1	0	0	0	0	0	0	0	0	0	0
Slightly reduced	4	4	6	4	7	9	6	6	1	1	0	0	0	0	0	0	0	0	0	0
Greatly reduced	0	0	0	0	2	0	4	4	5	1	5	6	4	2	3	0	0	0	0	0
None	0	0	0	0	0	1	0	0	3	7	8	7	5	7	5	8	5	5	6	6

**Table 2:** DMFT-Index (related to 32 teeth) and its components and ΔDMFT and ΔDFT, separated by mini mental state examination (MMSE) group and subgroup (control/experimental group) (evaluation time point—BL: baseline; Final (12 months after BL)) (Exp: experimental group; Con: control group) (MMSE group—noDem: no dementia; mCI: mild cognitive impairment; mDem: mild dementia; modDem: moderate dementia; sDem: severe dementia).

Item		noDem		mCI		mDem		modDem		sDem	
		Con (*n* = 12)	Exp (*n* = 12)	Con (*n* = 12)	Exp (*n* = 12)	Con (*n* = 10)	Exp (*n* = 13)	Con (*n* = 9)	Exp (*n* = 8)	Con (*n* = 5)	Exp (*n* = 6)
DMFT (related to 32 teeth)											
BL	Median (Range) Mean ± SD	29.5 (21–32) 28.5 ± 3.7	28 (18–32) 27.3 ± 3.8	28.5 (20–32) 28.4 ± 3.3	26 (17–32) 25.3 ± 4.4	27 (23–31) 26.7 ± 2.7	29 (20–32) 27.2 ± 3.6	30 (19–32) 28 ± 4.1	25.5 (20–32) 26.8 ± 4.5	18 (14–27) 19.4 ± 5.5	25.5 (17–31) 24.5 ± 4.7
Final	Median (Range) Mean ± SD	30 (21–32) 28.8 ± 3.6	28 (18–32) 27.7 ± 3.8	29 (20–32) 28.5 ± 3.3	26 (17–32) 25.6 ± 4.3	28 (23–32) 27.5 ± 2.9	29 (21–30) 27.4 ± 3.5	30 (19–32) 28.6 ± 4.4	26 (21–32) 27 ± 4.2	21 (14–27) 20.8 ± 5.1	26 (21–32) 26.2 ± 4
Δ DMFT (related to 32 teeth)											
Median (Range) Mean ± SD		0 (0–2) 0.3 ± 0.6	0 (0–2) 0.4 ± 0.7	0 (0–1) 0.1 ± 0.3	0 (0–2) 0.3 ± 0.7	0 (0–1) 0.2 ± 0.4	0.5 (0–2) 0.8 ± 0.9	0 (0–2) 0.6 ± 0.9	0 (0–1) 0.3 ± 0.5	0 (0–6) 1.4 ± 2.6	1 (0–4) 1.7 ± 1.5
DT											
BL	Median (Range)	0 (0–1)	0 (0–1)	0 (0–6)	0 (0–2)	0.5 (0–8)	0 (0–4)	1 (0–7)	0.5 (0–3)	1 (0–6)	0 (0–14)
Final	Median (Range)	0 (0–3)	0 (0–0)	0 (0–6)	0 (0–2)	1.5 (0–12)	0 (0–4)	1 (0–8)	0.5 (0–2)	1 (0–6)	5 (0–19)
MT											
BL	Median (Range)	21.5 (8–29)	17.5 (4–24)	16 (4–30)	12 (4–28)	13.5 (3–23)	10 (4–27)	13 (4–25)	12 (5–21)	7 (0–22)	8 (6–13)
Final	Median (Range)	21.5 (9–30)	18.5 (4–24)	16 (4–30)	11.5 (4–28)	14 (3–25)	10 (4–27)	11 (5–26)	12 (5–22)	9 (0–22)	7.5 (6–10)
FT											
BL	Median (Range)	6 (3–18)	10 (5–21)	10 (2–20)	12 (0–22)	13.5 (3–19)	16 (2–24)	10 (4–22)	13 (6–24)	8 (1–17)	16.5 (3–20)
Final	Median (Range)	6 (1–16)	10 (5–23)	10 (2–21)	13.5 (2–22)	12 (3–19)	16 (1–24)	9 (0–25)	13 (6–23)	12 (1–17)	14 (3–20)
DFT											
BL	Median (Range)	6 (3–18)	10 (5–21)	13 (2–21)	12.5 (1–22)	14 (4–20)	16 (2–24)	13 (5–26)	13.5 (7–24)	8 (5–18)	17.5 (8–20)
Final	Median (Range)	6 (2–16)	10 (5–23)	12.5 (2–22)	13.5 (2–22)	14.5 (3–20)	16 (3–24)	13 (6–25)	14 (8–24)	12 (5–18)	19.5 (12–22)
Δ DFT (related to 32 teeth)											
Median (Range) Mean ± SD		0 (−4–1) −0.6 ± 1.4	0 (−3–2) 0 ± 1.1	0 (−1–1) −0.8 ± 0.5	0 (0–2) 0.4 ± 0.7	0 (−5–4) 0.2 ± 2.9	0 (0–1) 0.2 ± 0.4	0 (−5–2) −0.2 ± 2.2	0 (−1–1) 0.1 ± 0.6	0 (0–4) 0.8 ± 1.8	2 (0–4) 2.2 ± 1.6

**Table 3:** Changes in the periodontal screening index (PSI—code 0: healthy; code 1/2: gingivitis/calculus present; code 3: moderate periodontitis; code 4: severe periodontitis) separated by mini mental state examination group (MMSE group) and subgroup (control and experimental group) at baseline and changes in PSI over time by MMSE group and subgroup (Con: control group; Exp: experimental group) (color coding—red: deterioration; orange: no change; green: improvement) (MMSE groups—noDem: no dementia; mCI: mild cognitive impairment; mDem: mild dementia; modDem: moderate dementia; sDem: severe dementia).

Degree of Dementia	Subgroup			PSI Code—Final				Total
				0	1–2	3	4	
noDem MMSE 28–30	Con	PSI Code—Baseline	1–2		2	1	0	3
			3		2	5	1	8
			4		0	0	1	1
		Total			4	6	2	12
	Exp	PSI Code—Baseline	1–2	0	1	1	0	2
			3	0	2	3	1	6
			4	1	1	0	2	4
		Total		1	4	4	3	12
mCI MMSE 25–27	Con	PSI Code—Baseline	1–2		2	1	0	3
			3		1	5	1	7
			4		0	0	2	2
		Total			3	6	3	12
	Exp	PSI Code—Baseline	3		3	2	0	5
			4		0	5	2	7
		Total			3	7	2	12
mDem MMSE 18–24	Con	PSI Code—Baseline	3			3	2	5
			4			2	3	5
		Total				5	5	10
	Exp	PSI Code—Baseline	3		0	6	0	6
			4		1	2	4	7
		Total			1	8	4	13
modDem MMSE 10–17	Con	PSI Code—Baseline	3			1	3	4
			4			3	1	4
		Total				4	4	8
	Exp	PSI Code—Baseline	3		1	2	4	7
			4		0	1	0	1
		Total			1	3	4	8
sDem MMSE < 10	Con	PSI Code—Baseline	4				2	2
		Total					2	2
	Exp	PSI Code—Baseline	3				2	2
			4				1	1
		Total					3	3

**Table 4:** Oral hygiene index (OHI) and its components (DI: debris index; CI: calculus index) and ΔOHI Calculus, ΔOHI Plaque, and ΔOHI Score, separated by mini mental state examination (MMSE) group and subgroup (control/experimental group) (evaluation time point—BL: baseline; Final (12 months after BL)) (Exp: experimental group; Con: control group) (MMSE groups—noDem: no dementia; mCI: mild cognitive impairment; mDem: mild dementia; modDem: moderate dementia; sDem: severe dementia).

	Item	noDem		mCI		mDem		modDem		sDem	
		Con (*n* = 12)	Exp (*n* = 12)	Con (*n* = 12)	Exp (*n* = 12)	Con (*n* = 10)	Exp (*n* = 13)	Con (*n* = 9)	Exp (*n* = 8)	Con (*n* = 5)	Exp (*n* = 6)
	OHI-Plaque (DI)										
BL		*n* = 12	*n* = 12	*n* = 12	*n* = 12	*n* = 10	*n* = 13	*n* = 9	*n* = 8	*n* = 3	*n* = 5
	Median (Range)	2 (0.7–5)	1.9 (1–2.5)	2.4 (0.8–4.5)	2.8 (1.3–4.2)	3.2 (1.8–3.8)	3.7 (2–5.5)	5 (2.4–6)	3.8 (2–4.8)	4.5 (2.5–5.3)	3.3 (3–4.8)
Final		*n* = 12	*n* = 12	*n* = 12	*n* = 12	*n* = 9	*n* = 13	*n* = 9	*n* = 8	*n* = 2	*n* = 3
	Median (Range)	1.1 (0.5–2)	0.3 (0–1.9)	1.3 (0.3–2)	0.5 (0.1–1.4)	2.2 (0.3–3)	2 (0–3)	2.1 (1.3–2.4)	2 (1.2–2.6)	2.1 (1.5–2.8)	2.4 (0.5–3)
Delta		*n* = 12	*n* = 12	*n* = 12	*n* = 12	*n* = 9	*n* = 13	*n* = 9	*n* = 8	*n* = 2	*n* = 3
	Median	−0.8	−1.3	−1.0	−2.0	−0.8	−2.1	−2.8	−1.9	−2.8	−0.8
	Range	−4–0.5	−2.3 to −0.1	−3.4 to −0.1	−3.5 to −0.7	−1.7–0.1	−4.1 to −0.2	−4–0	−3.4–0.4	−3 to −2.6	−2.8 to −0.5
	OHI-Calculus (CI)										
BL		*n* = 12	*n* = 12	*n* = 12	*n* = 12	*n* = 10	*n* = 13	*n* = 9	*n* = 8	*n* = 3	*n* = 4
	Median (Range)	1.5 (0–3)	1.6 (0.5–2.7)	2 (0–4.2)	1.9 (0.7–3.3)	2.8 (1.3–3.7)	3.2 (1.2–4.5)	4 (2.2–6)	2.7 (1.3–3.8)	3.3 (2.3–3.7)	3.3 (2.7–4)
Final		*n* = 12	*n* = 12	*n* = 12	*n* = 12	*n* = 9	*n* = 13	*n* = 9	*n* = 8	*n* = 2	*n* = 3
	Median (Range)	1.0 (0–1.8)	0.4 (0–1.6)	0.9 (0.5–1.7)	0.4 (0–0.9)	1.8 (0.3–3)	1.6 (0–3)	2 (1.1–2.2)	1.4 (0.4–2.5)	1.9 (1.4–2.4)	2.2 (0.9–3.3)
Delta		*n* = 12	*n* = 12	*n* = 12	*n* = 12	*n* = 9	*n* = 13	*n* = 9	*n* = 8	*n* = 2	*n* = 2
	Median	−0.6	−1.1	−1	−1.6	−0.8	−1.8	−2	−1.5	−1.6	−1.4
	Range	−1.7–1	−1.7–1.1	−3.5–0.7	−3 to −0.4	−1.6 to −0.3	−3.1 to −0.6	−4 to −0.2	−2.6–0.7	−2.3 to −1.0	−2.6 to −0.2
	OHI-Score										
BL		*n* = 12	*n* = 12	*n* = 12	*n* = 12	*n* = 10	*n* = 13	*n* = 9	*n* = 8	*n* = 3	*n* = 4
	Median (Range)	3.5 (1–7.7)	3.3 (1.8–4.7)	4.7 (1.7–8.7)	4.8 (2.7–7.5)	5.9 (3.2–7.4)	6.7 (3.2–9.5)	8.9 (4.7–12)	7 (3.3–7.8)	8.2 (4.8–8.7)	6.5 (6–8.8)
Final		*n* = 12	*n* = 12	*n* = 12	*n* = 12	*n* = 9	*n* = 13	*n* = 9	*n* = 8	*n* = 2	*n* = 3
	Median (Range)	2.1 (0.8–3.8)	0.7 (0–3.3)	2.2 (0.8–3.3)	1.1 (0.3–2.3)	4.1 (0.7–6)	3.1 (0–6)	4 (2.4–4.5)	3.6 (1.8–5.1)	4 (2.9–5.1)	4.6 (0.6–6.3)
Delta		*n* = 12	*n* = 12	*n* = 12	*n* = 12	*n* = 9	*n* = 13	*n* = 9	*n* = 8	*n* = 2	*n* = 2
	Median	−1.3	−2.2	−2.4	−3.5	−1.5	−3.9	−4.9	−3.5	−4.4	−3.1
	Range	−5.7–1.5	−4 to −0.2	−6.9–0.4	−6.1 to −1.1	−3.2 to −0.6	−7.2 to −1.2	−8 to −0.2	−5.2–1.1	−5.3 to −3.6	−5.4 to −0.7

## Data Availability

The data presented in this study are available from the corresponding author upon request. The data are not publicly available due to ethical reasons.

## Supplementary Materials

The following supporting information can be downloaded at: https://www.mdpi.com/article/10.3390/jcm11051356/s1, S1: Detailed description of measurements.
