# Supplementary material for: Impact of an Oral Hygiene Intervention in People with and without Dementia on Oral Health Parameters—Results from the Oral Health, Bite Force, and Dementia (OrBiD) Pilot Study
Source: J Clin Med. 2022 Mar 1;11(5):1356. doi: 10.3390/jcm11051356 (PMC8911423; doi:10.3390/jcm11051356)
Supplement: Supplementary file 1 [file jcm-11-01356-s001.zip › jcm-1608618-supplementary.pdf]

Supplementary Material S1

Detailed Description of the Measurements

The following parameters were evaluated.

- Sociodemographic data (age, sex, living situation (community-dwelling vs. LTCF)), the Mini-Nutritional Assessment (MNA) [26] (MNA score: normal: 24-30; at risk: 17-23.5; malnourished: <17), and the oral functional capacity (OFC) [27] were recorded for all subjects.
- DMF/T-Index [27]: The DMF/T index (D: decayed; M: missing; F: filled; T: teeth) is described as a measure of caries experience [28]. The World Health Organization (WHO) criteria were used for carious and filled teeth. Early stages of caries and caries stages preceding cavitation were excluded from registration as carious teeth. Older people often do not know why they lost a tooth many years ago. Therefore, it is difficult to distinguish whether a tooth was lost due to caries, periodontal disease, or trauma. Therefore, the DMF/T index useful in gerodontology as an exact measure of caries experience, but rather as an epidemiological description for older people [28, 29]. Therefore, missing teeth (MT) in this analysis included teeth missing due to caries, periodontal disease, or trauma. Since every tooth is valuable when considering older people, the DMF/T index in this study was related to 32 teeth (maximum value: 32).
- Periodontal Screening Index (PSI) [30]: PSI was recorded as the worst value per sextant (code 0: healthy; code 1: gingivitis without calculus/plaque or defective restorative margins; code 2: gingivitis with calculus and/or plaque and/or defective restorative margins; code 3: moderate periodontitis; code 4: severe periodontitis). A WHO periodontal probe was used for the evaluation. In the evaluation, code 1 and code 2 were added together as "gingivitis/calculus present". There were therefore only four categories in the evaluation: 0, 1-2, 3, and 4.
- Bleeding on probing (BOP): BOP (categorical variable: yes (+)/no (-)) indicates the presence of bleeding caused by gentle tissue manipulation at the depth of the gingival sulcus or the gingiva-to-tooth interface. If at least one site in the maxilla or mandible where probing depths have been measured with a periodontal probe (cf. Periodontal Screening Index) is positive (rating "yes"), BOP is rated as positive (yes, +) overall.
- Oral Hygiene Index (OHI) [31]: The OHI is used to evaluate and classify oral hygiene. It consists of two components: The Debris Index (DI) and the Calculus index (CI). The OHI is calculated according to the following rules. 1.) Only permanent, fully erupted teeth are evaluated. 2.) Wisdom teeth or teeth in infra-occlusion are not evaluated. 3.) The score is collected for both DI and CI, per sextant, separately for buccal and lingual tooth surfaces. The tooth surface most covered by plaque or sub/supragingival calculus in a sextant is evaluated. The OHI can achieve a score between 0 and 3 (0: no debris/calculus; 1: soft plaque/supragingival calculus covering no more than 1/3 of the tooth surface; 2: soft plaque covering more than 1/3 but no more than 2/3 of the tooth surface or supragingival tartar or small areas of subgingival calculus in the cervical area, or both; 3: soft plaque/supragingival calculus covering more than 2/3 of the tooth surface or a wide band of subgingival calculus in the cervical area. The calculation is made according to the following formula:

$$\text{DI} = \frac{\text{(Sum of all buccal debris scores in the upper and lower jaw) + (Sum of all lingual debris scores in the upper and lower jaw)}}{\text{Number of evaluated segments}}$$

$$\text{CI} = \frac{\text{(Sum of all buccal calculus scores in the upper and lower jaw) + (Sum of all lingual calculus scores in the upper and lower jaw)}}{\text{Number of evaluated segments}}$$

OHI = DI + CI

The higher the OHI (possible range 0-12), the poorer the oral hygiene. DI and CI can each take values from 0-6.
